# Quantitative expression analysis and prognostic significance of the *BCL2*-*associated X* gene in nasopharyngeal carcinoma: a retrospective cohort study

**DOI:** 10.1186/1471-2407-13-293

**Published:** 2013-06-18

**Authors:** Christos K Kontos, Ali Fendri, Abdelmajid Khabir, Raja Mokdad-Gargouri, Andreas Scorilas

**Affiliations:** 1 Department of Biochemistry and Molecular Biology, University of Athens, Panepistimiopolis, Athens, 15701, Greece; 2 Laboratory of Cancer Genetics and Production of Recombinant Proteins, Centre of Biotechnology of Sfax, B.P K.3038 Sfax Tunisia; 3 Habib Bourguiba University Hospital, B.P K.3038 Sfax Tunisia

**Keywords:** Head and neck cancer, Nasopharynx, Prognostic tumor biomarkers, Apoptosis, Quantitative real-time PCR

## Abstract

**Background:**

Nasopharyngeal carcinoma (NPC) is a highly metastatic epithelial malignancy showing high prevalence in Southeast Asia and North Africa. The *BCL2*-*associated X* (*BAX*) gene encodes the most important pro-apoptotic member of the BCL2 family. We have recently shown that *BCL2* and *BCL2L12*, two other members of the same apoptosis-related family, possess significant prognostic value in NPC. The objective of the current study was to analyze *BAX* mRNA expression in nasopharyngeal biopsies of NPC patients, and to assess its prognostic potential in this disease.

**Methods:**

Total RNA was isolated from 88 malignant and 9 hyperplastic nasopharyngeal biopsies, resected from Tunisian patients. After cDNA synthesis by reverse transcription of polyadenylated RNA, *BAX* mRNA expression was analyzed using a highly sensitive quantitative real-time polymerase chain reaction (qRT-PCR) method.

**Results:**

Lower *BAX* mRNA levels were detected in NPC biopsies than in hyperplastic nasopharyngeal samples. *BAX* mRNA expression status was associated with low tumor extent, negative regional lymph node status, and absence of distant metastases. Kaplan-Meier survival analysis demonstrated that patients with *BAX* mRNA-positive NPC have significantly longer disease-free survival (DFS) and overall survival (OS). In accordance with these findings, Cox regression analysis revealed that *BAX* mRNA expression can be considered as a favorable prognostic indicator of DFS and OS in NPC, independent of their gender, age, tumor histology, tumor extent, and nodal status. Furthermore, NPC patients without distant metastases are less likely to relapse when their primary tumor is *BAX* mRNA-positive, compared to metastasis-free patients with a *BAX*-negative nasopharyngeal malignancy.

**Conclusion:**

This is the first study examining the potential clinical utility of *BAX* as a prognostic tumor biomarker in NPC. We provide evidence that *BAX* mRNA expression can be considered as an independent favorable prognostic indicator of DFS and OS in NPC.

## Background

Apoptosis, the commonest mode of programmed cell death, plays a vital role in a wide variety of physiological processes by eliminating cells at the appropriate time and, therefore, controlling their number in development and throughout an organism’s life [1]. Defects in apoptotic cell death contribute utmost to the pathogenesis and progression of cancer by delaying or even preventing normal cell death, which results in abnormal cell accumulation [2,3]. The elucidation of the molecular machinery underlying apoptosis has uncovered the role of several proteins that are responsible, directly or indirectly, for the morphological and biochemical changes characterizing this phenomenon, such as chromatin condensation, DNA fragmentation, membrane blebbing and disruption of the maintained integrity of organelle structures along with formation of apoptosomes [4,5].

Perhaps the most known apoptosis-related family, BCL2, comprises many pro- and antiapoptotic proteins, showing partial structural similarity, as all of them contain at least one BCL2-homology domain (BH1, BH2, BH3, and/or BH4) [6,7]. The pro-apoptotic members of the BCL2 family, like BAX, BAD, BID and BCLX_S_, facilitate apoptosis, while the antiapoptotic members, such as BCL2, BCLX_L_ and BCLW, impede the apoptotic cell death machinery [8]. Interestingly, the relative ratios of pro- and antiapoptotic BCL2-family protein levels determine the sensitivity or resistance of cells to multiple apoptotic stimuli, including growth factor deprivation, hypoxia, irradiation, antineoplastic agents, oxidants, and Ca^2+^ overload [4,9,10]. In consistence with these findings, most BCL2-family members have been shown to constitute significant prognostic indicators for many solid tumors and blood malignancies, and/or putative biomarkers for monitoring of cancer patients’ response to chemotherapy [11,12].

BCL2-associated X (BAX) protein was the first apoptosis-inducing member of BCL2 family to be discovered [13]. Alternative splicing of the *BAX* gene produces four splice variants, each encoding a distinct protein isoform, namely BAX alpha, beta, delta, and sigma. Additionally, a non-coding transcript subjected to nonsense-mediated mRNA decay, named *BAX* variant epsilon, has been reported. The BAX alpha isoform bears the conserved BH1, BH2 and BH3 domains, and has a tertiary structure resembling that of BCLX_L_ and BCL2 [14]. The BH3 domain of BAX is essential for its homodimerization and its heterodimerization with BCL2 and BCLX_L_[15]. The formation of heterodimers between BAX and other members of the BCL2 family is involved in the regulation of apoptosis [16,17]. The high importance of BAX for the control of apoptotic cell death is reflected in the fact that cells overexpressing BAX show enhanced apoptosis whereas *BAX*-null cells are resistant to apoptosis [10,18]. *BAX* expression is also associated with tumor development and hematological malignancies [11,18].

A wide variety of tumors can arise in the nasopharynx, the most common being the nasopharyngeal carcinoma (NPC). NPC belongs to the family of lymphoepithelial carcinomas; these morphologically distinctive tumors can arise in a variety of sites, such as other head and neck mucosal sites, salivary gland, lung and thymus [19-21]. NPC is strongly associated with Epstein-Barr virus (EBV) infection, irrespectively of the ethnic origin of the patients, and represents one of the most frequent virus-related human malignancies, following liver carcinoma – associated with hepatitis B virus (HBV) and/or hepatitis B virus (HCV) presence – and cervix carcinoma, which shows a very strong association with human papillomavirus (HPV) infection [22]. Except for EBV infection, multiple other factors participate in the etiology of NPC, including genetic and epigenetic alterations as well as environmental factors, such as dietary habits [23-25].

NPC was initially reported in 1901 and clinically characterized in 1922 [21]. This malignancy shows a particular ethnic and geographic distribution [26]. Its highest incidence rates, varying between 15 and 50 per 100000 persons, are observed in South China and Southeast Asia, where the peak of incidence is at the age of about 50 years. NPC is also endemic in North Africa, showing a prevalence of 8 per 100000 persons and an additional minor peak of incidence occurring between the ages of 10 and 20 years, including about 25% of all NPC patients [27,28]. In Tunisia, particularly, NPC constitutes the most common type of head and neck cancer [29]. On the other hand, this malignancy is rather uncommon in the United States, accounting for 2% of all head and neck squamous cell carcinomas (HNSCCs), with an incidence of 0.5 to 2 per 100000 people. In addition, an intermediate incidence has been reported in Alaskan Eskimos and the Mediterranean Basin (North Africa, South Italy, Greece, and Turkey), ranging from 15 to 20 per 100000 persons [19].

Primary assessment of NPC is currently based on microscopic examination of cells and tissues. The strong association existing between NPC and EBV infection has pioneered a new paradigm of utilizing viral serological tests for cancer diagnosis and for screening in high-risk populations [30]. Furthermore, NPC is generally responsive to radiation therapy, and patients’ clinical outcome has significantly improved over the years, mostly due to refinements in staging and to improved therapy protocols [31]. Therapeutic decision-making is supported by a limited set of clinical, histological, and biological features. Notwithstanding this classification system has allowed important advances in cancer treatment, it is not always accurate [20].

To date, many efforts have been focused on the discovery of new biomarkers revealing the biological profile of each NPC case, therefore contributing to NPC diagnosis and prognosis, as well as to prediction of effective therapeutic strategies and monitoring of patients’ response to treatment. Several potential NPC biomarkers have been studied, including molecules implicated in pathways affecting key cellular properties, such as cell proliferation, apoptosis, invasion, and metastasis. Nevertheless, no established tissue molecular markers for NPC have been used so far in clinical practice; thus, the identification of novel prognostic and predictive biomarkers for NPC is a high necessity [32].

The aforementioned data prompted us to analyze *BAX* mRNA expression in 88 malignant and 9 hyperplastic nasopharyngeal biopsies using a highly sensitive quantitative real-time PCR (qRT-PCR) method that has previously been developed by members of our group, and to evaluate its potential prognostic significance and clinical application as a novel molecular tissue biomarker in NPC.

## Methods

### NPC patients and tissue specimens

Nasopharyngeal tissue biopsies were collected from 88 patients diagnosed with primary NPC and from 9 individuals with nasopharyngeal hyperplasia, at the Habib Bourguiba University Hospital of Sfax, in the South of Tunisia. All patients had not received any treatment prior to surgery. Sample collection took place between 2000 and 2007. Selection criteria for the specimens included the availability of sufficient tissue mass for RNA isolation. The selected patients represented approximately 45% of new NPC cases, diagnosed at the above institution during the accrual period, and the vast majority of them were EBV-positive. All biopsies were histologically confirmed by a pathologist. The clinical stage of nasopharyngeal biopsies was determined according to the tumor, node, and metastasis (TNM) classification system of the American Joint Committee on Cancer (AJCC) / Union for International Cancer Control (UICC), and the histological type was designated according to the World Health Organization criteria.

Biopsy samples were frozen in liquid nitrogen immediately after resection and stored at -80°C until further use. The current study was performed in accordance with the ethical standards of the Declaration of Helsinki in 1995 as revised in Tokyo in 2004, and was approved by the institutional Ethics Committee of CHU Habib Bourguiba (Sfax, Tunisia). Moreover, informed consent was obtained from all patients included in the study. Follow-up data included survival status (alive or deceased from NPC) and disease status (disease-free or recurrence/metastasis), along with dates of the events and cause of death.

### Human cell line culture

The human acute promyelocytic leukemia cell line HL-60 was maintained in RPMI 1640 medium, adjusted to contain 10% fetal bovine serum (FBS), 100 kU/L penicillin, 0.1 g/L streptomycin, and 2 mM L-glutamine. Cells were seeded at a concentration of 4×10^5^ cells/mL and incubated for 48 h at 37°C, in a humidified atmosphere containing 5% CO_2_, before being collected for further use.

### Isolation of total RNA and reverse transcription of polyadenylated RNA

Frozen hyperplastic and NPC tissue biopsies were pulverized with a scalpel on dry ice and total RNA was, then, isolated using the RNeasy Mini Kit (Qiagen Inc., Valencia, CA, US), according to the manufacturer’s instructions. Total RNA was assessed spectrophotometrically at 260 and 280 nm for its concentration and purity, and stored immediately at -80°C until further use.

First-strand cDNA was synthesized from polyadenylated RNA using a RevertAid™ First Strand cDNA Synthesis kit (Fermentas Inc., Glen Burnie, MD, US) in a 20-μL reverse transcription reaction mixture containing 2 μg of total RNA, following the manufacturer’s instructions.

### Quantitative real-time PCR

Since the four coding splice variants of the *BAX* gene encode proapoptotic protein isoforms, we chose to quantify them altogether, thus excluding from the quantification the non-coding splice variant of *BAX* which constitutes a nonsense-mediated mRNA decay candidate. Consequently, the primers were designed so as to generate a common (single) amplicon of 195 bp for all four protein-coding *BAX* transcripts. The sequences of the *BAX* primers were: 5’-TGGCAGCTGACATGTTTTCTGAC-3’ and 5’-TCACCCAACCACCCTGGTCTT-3’ , while the sequences of the *GAPDH* primers were: 5-ATGGGGAAGGTGAAGGTCG-3’ and 5’-GGGTCATTGATGGCAACAATATC-3’ , resulting in a 107-bp PCR amplicon. qRT-PCR was performed using the SYBR Green chemistry, according to the manufacturer’s instructions, in a 10-μL reaction mixture containing 10 ng of cDNA. PCR runs and melting temperature analysis were carried out in a 7500 Real Time PCR System (Applied Biosystems, Foster City, CA, US). Each reaction was performed in duplicate, in order to evaluate the reproducibility of data.

Calculations were made using the comparative C_T_ (2^-∆∆CT^) method, the application of which is based on the assumption that PCR efficiencies of the target gene and the endogenous control are very similar and quite 100% [33]. These prerequisites were checked in a validation experiment, as previously described [34]. *GAPDH* served as an endogenous control, while the leukemic cell line HL-60, in which *BAX* is expressed, was used as a calibrator, for the normalization of distinct PCR runs. Normalized results were expressed as arbitrary units (a.u.), which stand for the ratio of *BΑΧ* mRNA copies to 1000 *GAPDH* mRNA copies, calculated for each nasopharyngeal tissue biopsy, and in relation to the same ratio calculated for HL-60 cells.

### Statistical analysis

Owing to the non-Gaussian distribution of the expression levels of *BAX* in the NPC patients, analyses of the differences in *BAX* expression levels between malignant and non-malignant nasopharyngeal tissue biopsies were performed with the use of the non-parametric Mann–Whitney *U* test.

Transformation of continuous variables into discrete ones, usually dichotomous, is often very useful in laboratory medicine, as it enables stratification of patients into high versus low risk categories. To date, several methods are used to generate cutpoints, including biological determination, splitting at the median, and determination of the cutpoint that maximizes effect difference between groups. If the latter method (the so-called “optimal *P*-value” approach) is used, a dramatic inflation of type-I error rates can result [35]. A recently developed algorithm, X-tile, allows determination of an optimal cutpoint while correcting for the use of minimum *P*-value statistics [36]. As there are no established cutpoints available for *BAX* expression in NPC, the X-tile algorithm was used to generate an optimal cutoff for categorization of *BAX* mRNA expression. Thus, an optimal cutoff of 0.43 a.u. was generated, equal to the 40^th^ percentile.

According to the previously mentioned cutoff, *BAX* mRNA expression was classified as positive or negative, and associations between *BAX* expression status and other qualitative clinicopathological variables were analyzed using either the chi-square (Χ^2^) or the Fisher’s exact test, where appropriate.

Furthermore, univariate and multivariate Cox regression models were developed to evaluate the association between the prognostic markers and the relative risks for relapse and death of patients. Cox univariate regression analysis discloses the strength of the correlation between each clinicopathological parameter and disease-free survival (DFS) or overall survival (OS) [37]. The multivariate Cox regression models incorporated *BAX* mRNA expression and were adjusted for disease stage and histology. Survival analyses were also performed by constructing Kaplan-Meier DFS and OS curves, and their differences were evaluated using the log-rank (Mantel-Cox) test. The level of significance was defined at a probability value of less than 0.05 (*P* < 0.05).

## Results

### Clinical and biological features of NPC patients

Patients’ group consisted of 51 men and 37 women, and age at the time of diagnosis varied between 10.0 and 80.0 years, with a mean ± S.D. of 45.2 ± 17.9 and a median of 46.5. According to the AJCC classification system, 2 (2.3%) patient was diagnosed with stage I NPC, 12 (13.6%) with stage II, 22 (25.0%) with stage III, 12 (13.6%) with stage IV A, 13 (14.8%) with stage IV B, and 27 (30.7%) with stage IV C. Regarding the histology of the examined NPC biopsies, 46 out of 88 (52.3%) were of undifferentiated type and 42 (47.7%) were non-keratinizing carcinomas. Patients’ clinical and biological characteristics are summarized in Table 1.

**Table 1:** Clinical and biological characteristics of the NPC patients

**Number of patients**	**88**
**Gender** (Male/Female)	51/37
	**Median (range)**
**Age** (years)	46.5 (10.0 – 80.0)
**Disease-free survival** (months)	30 (2 – 90)
**Overall survival** (months)	36 (2 – 90)
	**Number of patients (%)**
**T – primary tumor extent**	
T0	0 (0%)
T1	8 (9.1%)
T2	25 (28.4%)
T3	19 (21.6%)
T4	36 (40.9%)
**N – regional lymph nodes**	
N0	14 (15.9%)
N1	14 (15.9%)
N2	28 (31.8%)
N3	32 (36.4%)
**M – distant metastasis**	
M0	61 (69.3%)
M1	27 (30.7%)
**TNM stage**	
I	2 (2.3%)
II	12 (13.6%)
III	22 (25.0%)
IV A	12 (13.6%)
IV B	13 (14.8%)
IV C	27 (30.7%)

### Quantitative *BAX* mRNA expression analysis in nasopharyngeal tissue specimens

**Figure 1:**
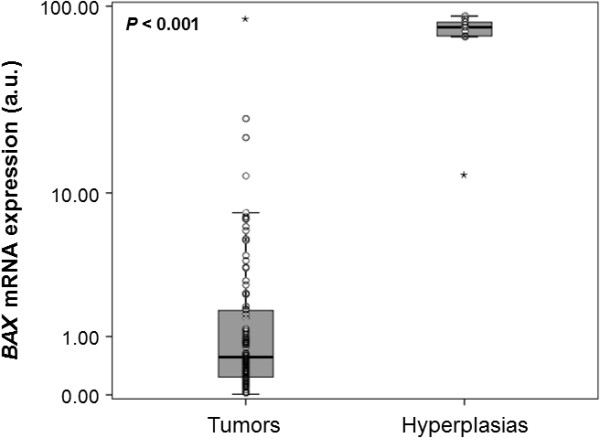
**Comparison of the distribution of*BAX*mRNA expression in malignant nasopharyngeal tumors and hyperplastic nasopharyngeal tissues.***BAX* transcripts encoding proapoptotic protein isoforms are far less abundant in NPC specimens than in hyperplastic tissue biopsies (*P* < 0.001). The *P* value was calculated using the non-parametric Mann–Whitney *U* test. The dark line near the middle of each box indicates the 50^th^ percentile (the median value) of each group, the bottom and top of each box represent the 25^th^ and 75^th^ percentile, respectively, and whiskers extend to 1.5 times the height of the box; the points represent outliers, while the asterisks show extreme outliers.

*BAX* mRNA levels in NPC biopsies ranged from 0.008 to 86.96 a.u. with a median of 0.57, whereas *BAX* mRNA expression in hyperplastic nasopharyngeal tissues varied between 12.58 and 88.77 a.u., with a median of 77.68 (Figure 1). Differences between these two groups were evaluated using the non-parametric Mann–Whitney *U* test, thus revealing a significant downregulation of *BAX* mRNA in biopsies collected from NPC patients (*P* < 0.001).

### Association of *BAX* mRNA expression status with patients’ clinicopathological variables

*BAX* mRNA expression was classified into two categories (positive or negative), as described in the “Methods” section. Therefore, of 88 NPC biopsies examined, 35 (39.8%) were classified as positive for *BAX* expression and 53 (60.2%) as negative. Table 2 presents the association between *BAX* mRNA expression status of the NPC biopsies with various clinicopathological parameters, as well as with patients’ gender and age. *BAX* positivity was more frequently observed in nasopharyngeal tumors of small tumor extent (T1 and T2) rather than in more extended NPC (T3 or T4; *P* = 0.014). Furthermore, regional lymph node status was found to be significantly associated with *BAX* mRNA expression status, as NPC patients with regional lymph node metastasis or unilateral metastasis in lymph nodes smaller than 6 cm in greatest dimension (N1) were more often *BAX*-positive, compared to patients with NPC classified as N2 or N3 (*P* = 0.024). Positive *BAX* mRNA expression status was also related to the absence of distant metastases (*P* = 0.018). Remarkable associations were not observed between *BAX* mRNA expression status and tumor histology, patients’ gender, or age at the time of diagnosis.

**Table 2:** **Relationships between*BAX*mRNA expression status and other clinicopathological variables**

			
		**Number of patients (%)**		
**Variable**	**Total**	***BAX*-negative**^**a**^	***BAX*-positive**^**a**^	***P*value**
**Cases**	88	35 (39.8)	53 (60.2)	
**Gender**				
Male	51	19 (37.3)	32 (62.7)	0.66^b^
Female	37	16 (43.2)	21 (56.8)	
**Age (years)**				
≤ 30	25	6 (24.0)	19 (76.0)	0.09^b^
> 30	63	29 (46.0)	34 (54.0)	
**Tumor histology**				
Undifferentiated	46	16 (34.8)	30 (65.2)	0.39^b^
Non-keratinizing	42	19 (45.2)	23 (54.8)	
**Tumor extent**				
T1 / T2	33	7 (21.2)	26 (78.8)	0.014^c^
T3	19	8 (42.1)	11 (57.9)	
T4	36	20 (55.6)	16 (44.4)	
**Regional lymph node status**				
N0	14	7 (50.0)	7 (50.0)	0.024^c^
N1	14	1 (7.1)	13 (92.9)	
N2	28	10 (35.7)	18 (64.3)	
N3	32	17 (53.1)	15 (46.9)	
**Distant metastasis**				
M0	51	19 (31.1)	42 (68.9)	0.018^b^
M1	27	16 (59.3)	11 (40.7)	

^a^Cutoff point: 0.43 a.u., equal to the 40^th^ percentile.

^b^Calculated by chi-square (Χ^2^) test.

^c^Calculated by Fisher’s exact test.

### *BAX* mRNA expression status as a favorable prognosticator for the disease-free survival of NPC patients

**Figure 2:**
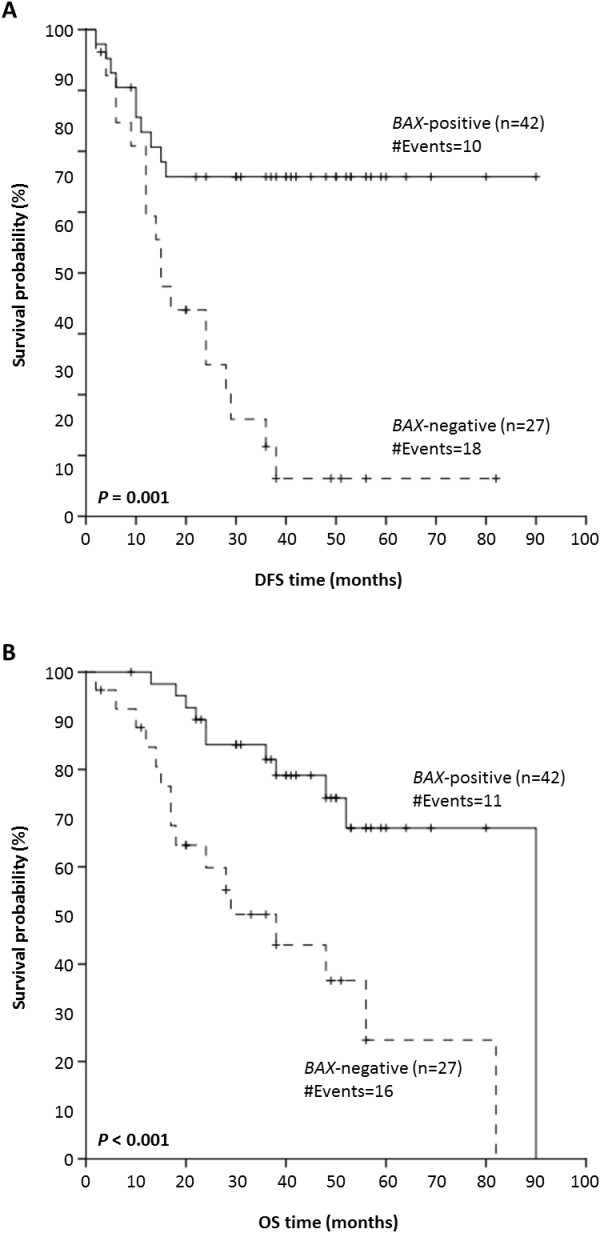
**Kaplan-Meier curves for disease-free survival (DFS) and overall survival (OS) of nasopharyngeal carcinoma (NPC) patients with*BAX*mRNA-positive and -negative nasopharyngeal tumors.***BAX* mRNA expression status possesses a favorable prognostic value in NPC, as patients with *BAX* mRNA-positive nasopharyngeal tumors have significantly longer DFS (*P* = 0.001) (**A**) and OS (*P* < 0.001) (**B**), compared to NPC patients with *BAX* mRNA-negative tumors.

Regarding DFS, out of 69 NPC patients for whom follow-up information was available, 28 patients (40.6%) relapsed during the respective follow-up periods. In Cox univariate regression analysis (Table 3), a 3.5-fold lower risk of recurrence was predicted for NPC patients bearing tumors with negative *BAX* mRNA expression status (hazard ratio [HR] = 0.28, 95% confidence interval [95% CI] = 0.13-0.62, *P* = 0.001). Therefore, in addition to tumor extent and TNM stage that were confirmed as significant predictors of DFS (*P* = 0.046 and *P* < 0.001, respectively), *BAX* gene expression at the mRNA level was shown to predict longer DFS in NPC. In order to evaluate *BAX* mRNA expression in terms of predicting survival outcome, we also performed Kaplan-Meier survival analysis. In accordance with the aforementioned results, Kaplan-Meier DFS curves illustrated that NPC patients with *BAX*-positive tumors had significantly longer DFS (*P* = 0.001), in comparison with those who had a *BAX*-negative malignant nasopharyngeal neoplasm (Figure 2A).

**Table 3:** ***BAX*mRNA expression and NPC patients’ survival**

	**Disease-free survival (DFS)**			**Overall survival (OS)**		
**Variable**	**HR**^**a**^	**95% CI**^**b**^	**p value**	**HR**^**a**^	**95% CI**^**b**^	**p value**
	**Univariate analysis**					
*BAX* mRNA expression						
Negative	1.00			1.00		
Positive	0.28	0.13 – 0.62	0.001	0.27	0.12 – 0.59	0.001
Gender (male / female)	1.42	0.66 – 3.09	0.37	1.81	0.79 – 4.18	0.16
Age (≤ 30 years / >30 years)	1.51	0.61 – 3.73	0.37	1.82	0.68 – 4.84	0.23
Tumor histology (undifferentiated / non-keratinizing)	0.59	0.28 – 1.25	0.17	0.72	0.33 – 1.57	0.41
Tumor extent (ordinal)	1.46	1.01 – 2.13	0.046	1.52	1.03 – 2.23	0.034
Regional lymph node status (ordinal)	1.34	0.91 – 1.98	0.14	1.32	0.90 – 1.94	0.16
TNM stage (ordinal)	2.79	1.82 – 4.26	<0.001	1.81	1.31 – 2.48	<0.001
	**Multivariate analysis**					
*BAX* mRNA expression^c^						
Negative	1.00			1.00		
Positive	0.35	0.15 – 0.86	0.022	0.33	0.13 – 0.84	0.020
Gender (male / female)	1.26	0.48 – 3.31	0.64	1.66	0.55 – 4.99	0.37
Age (≤ 30 years / >30 years)	1.45	0.53 – 4.01	0.47	1.88	0.59 – 5.95	0.28
Tumor histology (undifferentiated / non-keratinizing)	0.64	0.30 – 1.40	0.27	0.61	0.26 – 1.40	0.24
Tumor extent (ordinal)	1.26	0.76 – 2.06	0.37	1.34	0.79 – 2.29	0.27
Regional lymph node status (ordinal)	1.06	0.70 – 1.60	0.78	1.07	0.68 – 1.70	0.77
*BAX* mRNA expression^d^						
Negative	1.00			1.00		
Positive	0.67	0.29 – 1.56	0.35	0.36	0.16 – 0.84	0.018
Gender (male / female)	0.78	0.35 – 1.76	0.55	1.20	0.46 – 3.08	0.71
Age (≤ 30 years / >30 years)	1.68	0.66 – 4.31	0.28	2.18	0.76 – 6.24	0.15
Histology (undifferentiated / non-keratinizing)	0.95	0.43 – 2.12	0.90	0.64	0.27 – 1.50	0.30
TNM stage	2.60	1.68 – 4.04	<0.001	1.76	1.25 – 2.46	0.001

^a^Hazard ratio (HR), estimated from Cox proportional hazard regression model.

^b^Confidence interval of the estimated HR.

^c^Multivariate models were adjusted for gender, age, tumor histology, tumor extent, and regional lymph node status.

^d^Multivariate models were adjusted for gender, age, tumor histology, and TNM stage.

In the multivariate survival analysis (Table 3), *BAX* mRNA expression remained a statistically significant predictor of longer DFS in NPC, independent of patients’ gender, age, tumor histology, tumor extent, and regional lymph node status, as patients with *BAX* mRNA-positive tumors were more prone to relapse (HR = 0.35, 95% CI = 0.15–0.86, *P* = 0.022). However, when the TNM stage was included in the developed multivariate Cox regression model, *BAX* mRNA expression was not shown to have any additional prognostic impact.

### *BAX* mRNA expression status as an independent predictor of favorable overall survival of NPC patients

With regard to OS, out of 69 NPC patients for whom follow-up data were available, 27 patients (39.1%) died during the respective follow-up periods. As demonstrated by Cox univariate regression analysis (Table 3), NPC patients with *BAX* mRNA-positive nasopharyngeal tumors were at lower risk of death (HR = 0.27, 95% CI = 0.12–0.59, *P* = 0.001), compared to NPC patients whose biopsies were *BAX*-negative. Consequently, enhanced *BAX* mRNA expression seems to be a favorable prognosticator of OS, as well. Tumor extent and TNM stage were also significant prognosticators of OS (*P* = 0.034 and *P* < 0.001, respectively), as expected. In agreement with these results, Kaplan-Meier OS curves demonstrated that NPC patients with *BAX*-positive malignant neoplasms were more likely to succumb to their disease later than patients with *BAX*-negative nasopharyngeal tumors (*P* < 0.001) (Figure 2B).

In the multivariate Cox regression analysis (Table 3), *BAX* mRNA expression predicted a significantly favorable prognostic outcome (HR = 0.33, 95% CI = 0.13–0.84, *P* = 0.020), independent of patients’ gender, age, tumor histology, tumor extent, and regional lymph node status. More importantly, *BAX* mRNA expression retained its independent prognostic significance in NPC (HR = 0.36, 95% CI = 0.16–0.84, *P* = 0.018) even when the multivariate Cox regression model was adjusted for patients’ gender, age, tumor histology, and TNM stage.

### Prognostic value of *BAX* mRNA expression in NPC patients without distant metastases

**Figure 3:**
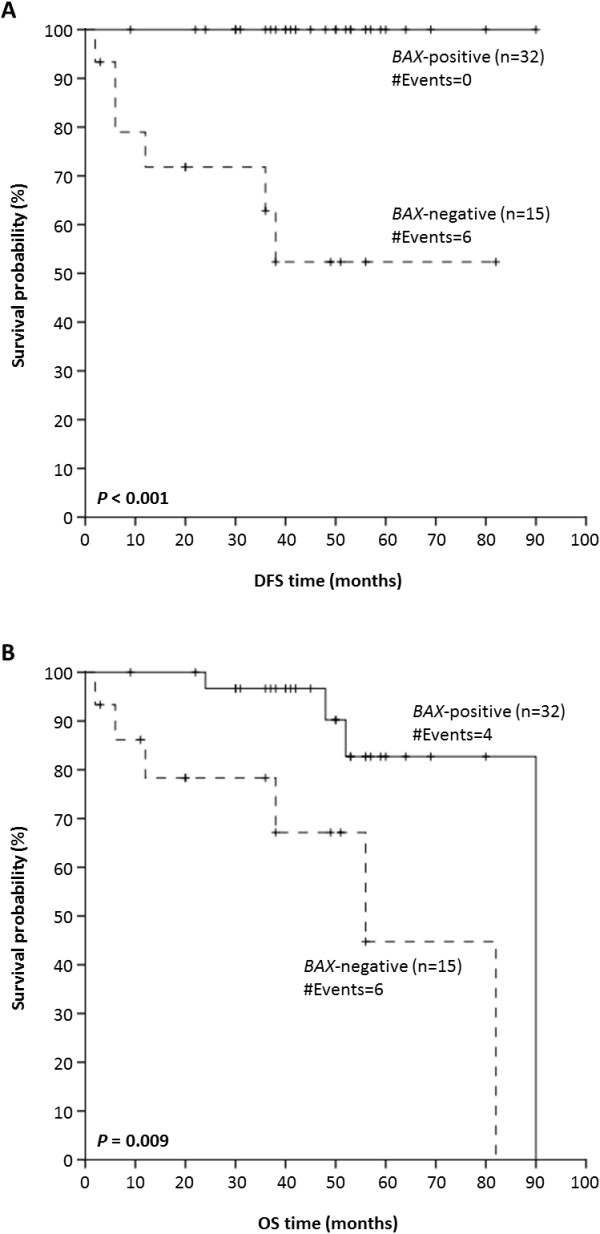
**Kaplan-Meier survival curves for NPC patients without distant metastases.***BAX* mRNA expression status possesses a favorable prognostic value in these patients, as patients with *BAX* mRNA-positive nonmetastatic nasopharyngeal tumors have a significantly higher probability of DFS (*P* < 0.001) (**A**) and OS (*P* = 0.009) (**B**) anytime, in comparison with metastasis-free patients bearing *BAX* mRNA-negative tumors.

Because metastasis-free (M0) patients are substantially different from those with metastases in distant organs (M1), in terms of their prognosis and postoperative treatment, Kaplan-Meier survival analysis was carried out to evaluate the effect of *BAX* mRNA expression on DFS and OS for metastasis-free NPC patients. As depicted in Figure 3, M0 patients with *BAX*-positive malignant nasopharyngeal tumors had more favorable DFS and OS rates than did M0 patients with *BAX*-negative malignancies (*P* < 0.001 and *P* = 0.009, respectively).

## Discussion

Although radiotherapy and chemotherapy have improved survival rates of NPC patients [38], the prognosis for metastatic NPC remains poor, even when patients are treated with combined radiotherapy and chemotherapy; notably, recurrence rates are very high, up to 82% [39]. Unfortunately, the majority of NPC patients are diagnosed at an advanced stage, mostly due to the non-specific symptoms of the disease, to the delay in undergoing treatment after the onset of symptoms, and to the difficulty of being subjected to a thorough nasopharyngeal examination [40]. Consequently, supplementary work is needed to elucidate the molecular alterations being involved in NPC carcinogenesis, to discover reliable tissue biomarkers for NPC, and to develop novel, effective targeted therapeutic strategies against NPC.

The prognostic and predictive value of several genes and/or proteins in NPC has been assessed during the past decades. Among them, factors related to key cell functions such as cell cycle, growth, proliferation, and apoptosis, have drawn the most attention. A recent study revealed the prognostic significance of *PTGS2* (*COX2*) mRNA expression; high levels of *PTGS2* mRNA are associated with lymph node metastasis in NPC patients [41]. Furthermore, aberrant promoter methylation of *RASSF1*, *RARB*, and *DAPK1* have been linked to advanced NPC, characterized by advanced stage and positive lymph nodes [42]. Guo *et al*. have noticed a remarkable decrease of *DROSHA* and *DICER* mRNA levels in NPC compared to healthy control samples, and shown that they are significantly related to shorter progression-free survival (PFS) and OS of NPC patients [43]. Cytokeratin-18 (KRT18), the dysregulation of which is supposed to play an important role in nasopharyngeal carcinogenesis, constitutes another potential biomarker for the differentiation and prognosis of NPC [44]. Furthermore, cytoplasmic heterogeneous nuclear ribonucleoprotein K (HNRNPK) and thymidine phosphorylase (TYMP) have been suggested as independent indicators of prognosis in NPC [45]. Additionally, low expression of the breast cancer metastasis suppressor 1 (*BRMS1*) gene – the product of which is a component of the mSin3a family of histone deacetylase (HDAC) complexes – was shown to enhance NPC metastasis both *in vitro* and *in vivo*, and to be associated with poor survival of NPC patients [46]. Numerous studies have also demonstrated the correlation of COP9 constitutive photomorphogenic homolog subunit 5 (COPS5) overexpression with poor prognosis in NPC [47].

The prognostic potential of apoptosis-signaling proteins such as BCL2-family members has been well documented so far in numerous human malignancies, including leukemias and lymphomas, hormone-dependent tumors, colorectal cancer, and head and neck cancer [11,48]. It has even been suggested that systems analysis of BCL2 protein family interactions can establish a model to predict chemotherapy response [49]. Regarding NPC, we have recently shown that mRNA expression status of *BCL2* is strongly associated with lymph node involvement and presence of distant metastases in patients, and that it may therefore represent a novel unfavorable and independent tumor biomarker of this malignancy [50]. In accordance with these previous findings, overexpression of the antiapoptotic BCL2 protein, a modulator of lymph node metastasis of NPC cells [51], predicts advanced-stage NPC with satisfactory accuracy [52]. Surprisingly enough, another study showed a better clinical outcome for BCL2-positive NPC patients. A putative explanation for this seemingly contradictory finding could be the tumor histological type, as BCL2 protein expression is significantly associated to undifferentiated NPC [53]. Another reason could be the fact that BCL2 can sometimes exert a proapoptotic function. Although BCL2 at low expression levels in gliomas is antiapoptotic, high levels of BCL2 facilitate FASLG-mediated apoptosis in this cancer type [54]. Furthermore, cleavage of BCL2 by caspases enhances the activation of downstream caspases and contributes to amplification of the caspase proteolytic cascade [55]. BCL2 can also be converted into a proapoptotic protein by the nuclear receptor subfamily 4, group A, member 1 (NR4A1) [56,57]. According to our previously published results, *BCL2L12* mRNA expression is also associated with unfavorable prognosis in NPC patients and may represent a novel molecular biomarker for the prediction of short-term relapse in NPC. Moreover, *BCL2L12* overexpression is likely to account for resistance of NPC patients with advanced-stage disease to chemotherapeutic and irradiation treatment [58].

BAX is a key proapoptotic molecule in the intrinsic apoptotic pathway, as its insertion into the mitochondrial membrane triggers the release of cytochrome C into the cytosol, leading to caspase activation and to subsequent cell apoptosis [59]. In the current study, expression analysis of the proapoptotic *BAX* gene in NPC and in hyperplastic nasopharyngeal tissue biopsies revealed a significant downregulation of *BAX* mRNA levels in the former, in comparison to the latter. This finding along with the fact that the BAX inhibitor TMBIM6 mediates resistance to apoptosis in human NPC cells [60] directly imply that dysregulation of BAX is implicated in nasopharyngeal carcinogenesis. Furthermore, our results demonstrate that *BAX* mRNA expression is significantly associated with various clinicopathological parameters, including primary tumor extent, regional lymph node status, and presence of distant metastases. In particular, *BAX* mRNA was diminished in advanced-stage (T3 and T4) nasopharyngeal tumors and/or metastatic tumors, accompanied either by a number of positive regional lymph nodes only, or also by infiltration of distal organs. At this point, it should be noted that advanced stages of the disease have been correlated to high plasma/serum EBV DNA titers [61]. The apparent downregulation of BAX is probably attributed to the EBV latent membrane protein 1 (LMP1). This antiapoptotic protein was shown to protect B-cells from apoptosis by inhibition of *BAX* transcription through activation of the NFKB (P50/P65 heterodimer), which reduces BAX promoter activity [62].

Our survival analysis uncovered the potential of *BAX* mRNA expression status as a strong favorable predictor of DFS and OS in NPC. Cox proportional hazard regression analysis confirmed that *BAX* is a significant independent prognostic factor in NPC, as patients with *BAX*-positive nasopharyngeal tumors were at a reduced risk of relapse and death, independently of their gender, age, tumor histology, tumor extent, and nodal status. Perhaps even more important was the finding that NPC patients without distant metastases are less likely to relapse when their tumor is *BAX* mRNA-positive, compared to metastasis-free patients with a *BAX*-negative nasopharyngeal malignancy. To the best of our knowledge, this is the first study examining the prognostic value of *BAX* in NPC. However, the favorable prognostic role of BAX has already been shown in other head and neck malignancies, such as oral squamous cell carcinoma and esophageal cancer. In particular, high BAX expression is significantly associated with elevated MKI67 (proliferation-related Ki-67 antigen) expression, suggesting that increased proliferation might lead to an improved response to radiotherapy in patients with elevated BAX protein levels [63]. Furthermore, the BAX/BCL2 ratio was shown to predict response to neoadjuvant radiochemotherapy in patients with advanced squamous-cell esophageal cancer [64].

## Conclusion

To the best of our knowledge, this is the first time that this gene is studied in NPC. Our results suggest that *BAX* mRNA expression is related to favorable prognosis in NPC and that it may represent a novel, useful tissue biomarker for the prediction of short-term relapse and overall survival of NPC patients. *BAX* overexpression may also account for sensitization of NPC patients with advanced-stage disease to chemotherapeutic and irradiation treatment. Undoubtedly, future studies are needed to elucidate the functional role of BAX in nasopharyngeal tumors. Moreover, it would be very tempting to develop an ELISA-based methodology for the quantification of BAX protein levels in NPC specimens, in order to investigate the putative prognostic value of the BAX protein in NPC and to evaluate further the potential of this molecular biomarker in NPC patients. Differences in quantities of apoptosis-related proteins including BAX could also be exploited in the development of multivariate models aiming at predicting patients’ response to chemotherapy. Hence, NPC patients could benefit from tailor-made chemotherapeutic treatment.

## Abbreviations

BAX: BCL2-associated X; BH: BCL2-homology; NPC: Nasopharyngeal carcinoma; EBV: Epstein-Barr virus; HBV: Hepatitis B virus; HCV: Chepatitis C virus; HPV: Human papillomavirus; HNSCC: Head and neck squamous cell carcinoma; qRT-PCR: Quantitative real-time polymerase chain reaction; TNM: Tumor node, metastasis; AJCC: American Joint Committee on Cancer; UICC: Union for international cancer control; cDNA: DNA complementary to RNA; RNase: Ribonuclease; oligo(dT): Oligodeoxythymidine; DTT: Dithiothreitol; dNTP: Deoxyribonucleoside triphosphate; GAPDH: Glyceraldehyde-3-phosphate dehydrogenase; a.u: Arbitrary units; DFS: Disease-free survival; OS: Overall survival; HR: Hazard ratio; CI: Confidence interval; HNRNPK: Heterogeneous nuclear ribonucleoprotein K; TYMP: Thymidine phosphorylase; BRMS1: Breast cancer metastasis suppressor 1; HDAC: Histone deacetylase; COPS5: Constitutive photomorphogenic homolog subunit 5; FASLG: Fas ligand; NR4A1: Nuclear receptor subfamily 4 group A, member 1.

## Competing interest

The authors declare that they have no competing interests.

## Authors’ contributions

CKK carried out part of the experimental work, collected and analyzed data, performed the statistical analysis, interpreted the results, and drafted the manuscript. AF carried out most of the experimental work and drafted the manuscript. AK designed the study, collected patients’ material and follow-up data. RMG designed the study and revised critically the manuscript. AS conceived of the study, coordinated the study, and revised critically the manuscript. All authors read and approved the final manuscript.

## Pre-publication history

The pre-publication history for this paper can be accessed here:

http://www.biomedcentral.com/1471-2407/13/293/prepub
